# Supplementary material for: Annual report text’s positive tone and corporate green innovation: Evidence from China
Source: PLoS One. 2024 Jul 29;19(7):e0306184. doi: 10.1371/journal.pone.0306184 (PMC11285926; doi:10.1371/journal.pone.0306184)
Supplement: Attachment — Submitted filename: Response to Reviewers.docx [file pone.0306184.s002.docx]

Dear Editors and Reviewers:

Thank you very much for your comments and professional advice concerning our manuscript entitled ''**Annual report text's positive tone and corporate green innovation: Evidence from China**'' (**ID: PONE-D-24-12298**). Those comments are all valuable and helpful for revising and improving our paper. We have studied the comments carefully and have revised our manuscript. Words in **blue color** are the changes we have made in the revised manuscript. In addition, since the sample time has been updated to 2022, the data in the table in the revised manuscript has also been updated accordingly. Now we respond to your comments **point by point** and highlight the changes in the revised manuscript. We sincerely hope that you find our responses and modifications satisfactory. The responses to your comments are listed as follows:

**Reviewer # 1:**

**1.Comment:** Equation (2): There are spelling errors in Equation (2), such as "cashflow" and "growth," and there are missing subscripts, such as "it."

**1. Response:** Thank you for your comments and suggestions, we have modified the spelling errors in Equation 2, the revised Equation 2 as follows:

${Green}_{i,t}=\alpha_{0}+\alpha_{1}{Tone}_{i,t}+\alpha_{2}S{ize}_{i,t}+\alpha_{3}{Lev}_{i,t}+\alpha_{4}{ROA}_{i,t}+\alpha_{5}{Cashflow}_{i,t}+\alpha_{6}{Growth}_{i,t}+\alpha_{7}{Board}_{i,t}+\alpha_{8}{Indep}_{i,t}+\alpha_{9}{Top1}_{i,t}+\alpha_{10}{ListAge}_{i,t}+\alpha_{11}{Dual}_{i,t}+\alpha_{12}{SOE}_{i,t}+\alpha_{13}{Year}_{i,t}+\alpha_{14}{Ind}_{i,t}+\varepsilon_{i,t} (2)$(**Line 209-211 on page8)**

**2. Comment:** Economic Effect Analysis: Please consider incorporating an economic effect analysis similar to the approach followed by Wang et al. (2024), taking into account multiple enterprise performance indicators.

**2. Response:** Thank you for your suggestion. according to your suggestion, we have adopted an economic effects analysis method similar to that employed by Wang et al. (2024), and have considered multiple firm performance indicators simultaneously. The revised section as follows:

''Since enterprises have invested a lot in the early stage of implementing a green innovation strategy, its economic benefits may not be reflected in the short term. Therefore, for corporate value indicators, following Wang et al. (2024), we adopt Tobin’s Q ratio (*TobinQ*) and the Price-to-Book ratio (*PB*) to measure, focusing on the impact on the long-term value of enterprises, and constructs the following Equation (4) to test the effect of positive tone of annual report text and green innovation on corporate value. The other control variables in Equation (4) are consistent with Equation (2), focusing on the coefficient of cross-term *Tone*Green*.

The regression results are shown in Table 12. In column (1) and (2), the coefficient of *Tone*Green* is significantly positive at a 1% level, indicating that the positive tone of the annual report text has a positive impact on corporate green innovation and can improve corporate value. ''(**Line 418-420; 424 on page21)**

**3. Comment:** Endogeneity Issue: I recommend employing the Generalized Method of Moments (GMM) to address the endogeneity issue present in the study.

**3 Response:** Your comments have been very helpful to us, according to your suggestion, we have adopted the instrumental variable method to solve the endogeneity problem, the revised section as follows:

''*4.5.6 Instrumental variable method*

Considering that there may be a reverse causal relationship between the positive tone of annual report texts and green innovation, following Li and Jiang (2020), we select the mean value of the positive tone of texts in the same year and industry as the instrumental variable (*Tone_m*) and adopt the two-stage least squares method (IV-2SLS).

The regression results are shown in Table 8. The test results of weak instrumental variables show that the F value is 3054.94, which is significantly greater than the empirical value 10. The instrumental variables satisfy the correlation and are strong instrumental variables. The regression coefficient of *Tone* is still significantly positive, which once again verifies the positive tone of the annual report text to promote enterprises' green innovation.'' (**Line 309-319 on page15**)

**Table 8	Instrumental variable method**

| Variable | *Green* | |
| --- | --- | --- |
|  | (1) | (2) |
| *Tone_m* | 1.3852^**^ |  |
|  | (2.0597) |  |
| *Tone* |  | 1.7996^**^ |
|  |  | (2.0609) |
| *Size* | 0.1372^***^ | 0.1364^***^ |
|  | (33.8924) | (33.2899) |
| *Lev* | 0.1407^***^ | 0.1352^***^ |
|  | (5.6741) | (5.4336) |
| *ROA* | 0.4021^***^ | 0.3346^***^ |
|  | (5.5959) | (4.2046) |
| *Cashflow* | 0.0840 | 0.0920 |
|  | (1.3763) | (1.5049) |
| *Growth* | -0.0509^***^ | -0.0554^***^ |
|  | (-5.3560) | (-5.6942) |
| *Board* | 0.0765^***^ | 0.0742^***^ |
|  | (3.1126) | (3.0151) |
| *Indep* | 0.0009 | 0.0009 |
|  | (1.1232) | (1.1035) |
| *Top1* | -0.0005^*^ | -0.0005^*^ |
|  | (-1.7100) | (-1.6592) |
| *ListAge* | -0.0812^***^ | -0.0792^***^ |
|  | (-13.9104) | (-13.2109) |
| *Dual* | 0.0177^**^ | 0.0171^*^ |
|  | (2.0134) | (1.9424) |
| *SOE* | 0.0814^***^ | 0.0763^***^ |
|  | (8.1450) | (7.2863) |
| _cons | -2.9496^***^ | -2.9495^***^ |
|  | (-9.2057) | (-9.2108) |
| Year fe | Yes | Yes |
| Ind fe | Yes | Yes |
| F-value | 3054.94 | |
| *N* | 34799 | 34799 |
| r2 | 0.1754 | 0.1764 |

Note: ***p<0.01, **p<0.05, *p<0.1 are significant at the level of 1%, 5% and 10%, respectively.''

**4. Comment:** Theoretical Hypotheses: Please include a discussion in the theoretical hypotheses section regarding how the positive tone in annual reports can positively influence enterprises' green innovation through the mechanisms of alleviating financing constraints and enhancing external attention, as suggested by previous literature (e.g., Wang et al., 2024).

**4. Response:** Thank you for your suggestion, according to your suggestion, we have added relevant literature research to the revised manuscript. We hope that the current theoretical analysis is more reasonable, and the revised section is as follows:

'' This enables investors to have a deeper understanding of the economic conditions of enterprises and increase their investment in enterprises (Feldman et al., 2010; Liu and Wang, 2021), to reduce financing constraints (Qiu and Yang, 2021; Wang et al., 2024), effectively solve the financing dilemma of enterprises' green innovation, and improve the willingness of enterprises to green innovation. '' (**Line 131 on page 5)**

'' In the current wave of green and low-carbon development, the public's environmental demands for green and low-carbon, ecological, and environmental protection are important driving forces for enterprises to carry out green innovation and development and achieve green transformation (Wang et al., 2024). Therefore, under the supervision and pressure of external analysts, enterprises will actively implement green innovation activities to meet the demands of the public. (Chen et al., 2018; Wang et al., 2024). '' (**Line 139-141 on page 5)**

The above contents are our responses to your valuable questions or suggestions. Thank you very much for your patience! Your patience and high-quality advice are helpful to improve our paper.

**Reviewer # 2:**

**1. Comment:** The introduction mentions, "Corporate managers may deliberately use a positive tone to mislead investors to cover up the poor performance of enterprises in environmental and social domains." Is this the motivation for the research? Has the manuscript addressed this issue? If so, how?

**1. Response:** Thank you for pointing out this issue. as for the role of the positive tone of the annual report text, the academic community has drawn two opposite views. There are positive and negative views on the role of positive tone in the annual report text. Then, when it comes to the impact on green innovation, does positive text intonation play a positive information incremental effect or a negative information masking effect? This is also the motivation of this study. After empirical testing of samples from 2010 to 2022, this study concluded that positive intonation can promote enterprises to carry out green innovation behaviors, supporting the information incremental view. And further research shows that positive tone can improve the level of green innovation and enterprise value at the same time, which further proves the positive role of positive tone.

**2. Comment:** The language of the manuscript requires improvement. For example, sections such as "Section 2 literature review, theoretical analysis and hypothesis" and "Section 5 mechanism test and heterogeneity analysis" lack necessary sentence components.

**2. Response:** We feel so sorry for the mistakes in the manuscript and inconvenience they caused in your reading. The language in our revised manuscript has been professionally edited and verified by a native English speaker. Thank you very much for your precious comments. In addition, we have improved our presentation by clearly labeling equations, tables, sections, and subsections. We really hope that the flow and language level have been substantially improved.

**3. Comment:** The research hypotheses presented should be consistent with the conclusions that the paper aims to verify; therefore, one hypothesis should be removed.

**3. Response:** Thank you very much for your suggestions. Please allow us for some explanation about this problem. There are positive and negative views on the role of positive tone in the annual report text. The research perspective of this paper is green innovation, from the theoretical level, we can deduce two opposite logical paths. Before the empirical test, we do not know whether it is positive or negative, and only through the subsequent empirical test can we reach the final conclusion. Therefore, we put forward two competing hypotheses in the theoretical analysis and hypothesis part.

**4. Comment:** The author seems to have a non-rigorous attitude towards academic writing, as seen in Section 3, "Following prior studies (),".

**4. Response:** Thank you for pointing out this issue. We feel so sorry for the mistakes in the manuscript. We have revised this question, as well as other related questions throughout the manuscript. The modified section is as follows:

'' Following prior studies (Xu et al., 2020; Yu, 2022)'' (**Line 543-551 on page 24**).''

**5. Comment:** Why does the data only extend to 2020? What is the reason for this?

**5. Response:** Thank you for pointing out this issue. We have updated the sample data to 2022.

**6. Comment:** The practice of adding 1 to the data before taking logarithms, which has been considered as distorting the data and regression results, should be reevaluated. Why not consider using raw data for regression?

**6. Response:** Thank you for pointing out this question. After descriptive statistics, we found that the green innovation data was obviously skewed to the right. Therefore, we followed the practice of previous studies and processed the green innovation data with logarithm after +1. At the same time, in the robust test part, we also used the original data of green innovation to conduct a re-regression test, and the research conclusion remained unchanged.

**7. Comment:** Have macroeconomic variables not been considered among the control variables?

**7. Response:** We did not take macro variables as control variables for two reasons: First, because the problem we studied was at the micro enterprise level, it was more affected by corporate governance and financial characteristics. Second, the selection of control variables in this study also follows the practice of previous studies.

**8. Comment:** There are unrecognizable symbols in Equation (2); please verify.

**8. Response:** Thank you for pointing out this question. The revised Equation (2) is as follows:

${Green}_{i,t}=\alpha_{0}+\alpha_{1}{Tone}_{i,t}+\alpha_{2}S{ize}_{i,t}+\alpha_{3}{Lev}_{i,t}+\alpha_{4}{ROA}_{i,t}+\alpha_{5}{Cashflow}_{i,t}+\alpha_{6}{Growth}_{i,t}+\alpha_{7}{Board}_{i,t}+\alpha_{8}{Indep}_{i,t}+\alpha_{9}{Top1}_{i,t}+\alpha_{10}{ListAge}_{i,t}+\alpha_{11}{Dual}_{i,t}+\alpha_{12}{SOE}_{i,t}+\alpha_{13}{Year}_{i,t}+\alpha_{14}{Ind}_{i,t}+\varepsilon_{i,t} (2)$(**Line 209-211 on page8)**

**9. Comment:** The descriptive statistics show that more than half of the data on green technology innovation are zeros. Is this a true reflection of the situation?

**9. Response:** Thank you for pointing out this issue. This is a true reflection of the situation, and previous studies have come to the same result.

**10. Comment:** Is there a basis for the calculation of Tone2?

**10. Response:** Thank you very much for pointing out our problem. For the measurement of *Tone2*, the intonation library built is the same as *Tone*, but different calculation formulas are adopted. The specific formula is shown in Equation (3). Therefore, in the original manuscript, we simplified the process and did not describe the construction of the intonation library again. We have re-described the *Tone2* measurement process in detail in the revised manuscript, hoping that our revisions can make the manuscript more clear. The revised contents are as follows.

''To overcome the influence of the measurement bias of explanatory variables, Following Xie and Lin (2015), first of all, we take positive color words (" positive, "high quality," "promotion," etc.) and negative color words (" low, "unfavorable," "downward," etc.) as a list of positive and negative words respectively. Then, we used Python programming to count the positive and negative words in the MD&A of the annual report to obtain the total number of positive and negative words. Finally, refering to the practice of Li and Jiang (2020), we use the Equation (3) to calculate the report text's positive tone (*Tone2*).'' (**Line 265-269 on page12)**

**11. Comment:** The section on research hypotheses could benefit from additional theoretical discussion related to the mechanism section.

**11. Response:** In the part of theoretical analysis and hypothesis, we put forward two opposing hypotheses based on the two logic chains of information increment view and impression management view. But which hypothesis is valid? After the empirical test, our conclusion validates the information increment view, that is, the positive tone of the annual report text can promote the green innovation behavior of enterprises.Therefore, in the latter part of the mechanism test, we need to find out the channel through which the positive tone of the annual report text promotes the green innovation of enterprises under the theoretical framework of the incremental view of information (theoretical analysis and hypothesis).

**12. Comment:** The author needs to discuss the rationale and significance behind analyzing heterogeneity.

**12. Response:** Thank you for your suggestion, according to your suggestion, we rewrote the heterogeneity analysis section. The rewritten content is as follows:

''*5.2.1 The* *influence of economic policy uncertainty*

China, transitioning from high-speed economic growth to high-quality development, faces significantly more risks and challenges, and its economic development has significant destabilizing and uncertain factors. To cope with the downward economic pressure brought about by risks and challenges, China has continuously carried out macroeconomic policy reform and innovation, and the uncertainty of economic policy has increased significantly (Chen and Chen, 2018), which not only directly affects the efficiency and effect of the macroeconomic operation, but also spreads to micro-enterprises through the capital market mechanism. Finally, it will become an important external force affecting the green innovation of micro-enterprises. The rise of economic policy uncertainty will exacerbate market information asymmetry and increase enterprises' financing difficulty, thus inhibiting enterprises' green innovation (Drobetz et al., 2018; Arellano et al., 2019; Song et al., 2019). The positive tone of the annual report can reduce the information asymmetry between enterprises and external markets, thus easing the financing constraints of enterprises and providing funds for enterprises to carry out green innovation. Therefore, we predict that the positive tone of the annual report text will promote corporate green innovation more significantly in listed companies with high economic policy uncertainty.

Following Ding and Wang (2021), we adopted the uncertainty index of China's economic policy constructed by Baker et al. (2016) based on the South China Morning Post, a news report retrieval platform, through keyword search. Then, the logarithm of the arithmetical mean of the economic policy uncertainty index for each month of the year is further calculated, and the logarithm of the monthly indicator is converted into the logarithm of the annual indicator and then divided by 100 for standardization processing as the final proxy indicator (*EPU*) of economic policy uncertainty.

We use the mean value of the economic policy uncertainty index of listed companies as the grouping criterion. If the economic policy uncertainty faced by enterprises is lower than the mean value of the index, they are divided into the low economic policy uncertainty group; if the economic policy uncertainty is higher than the mean value of the index, they are divided into the high economic policy uncertainty group. If the positive Tone coefficient of the annual report is significantly different in different subgroup samples, it indicates that the positive tone of the annual report has different effects on the green innovation of enterprises under different external environments.

The regression results are shown in Table 10. When economic policy uncertainty is high, the coefficient of *Tone* is 2.5714, which is significant at the 1% level. When economic policy uncertainty is low, the coefficient of *Tone* is 0.9124, also significant at the 1% level. However, when we further used the Bootstrap sampling method to test the significance of inter-group coefficient differences, the corresponding P-value was 0.000, indicating that when the economic policy uncertainty was higher, the positive tone of the annual report had a more obvious role in promoting corporate green innovation. This test result further confirmed the information incremental effect of positive tone of the text. (**Line 347-382 on page18-19)**

**Table 10	The influence of economic policy uncertainty**

| Variable | *Green* | |
| --- | --- | --- |
|  | (1)  *EPU_H* | (2)  *EPU_L* |
| *Tone* | 2.5714^***^ | 0.9124^***^ |
|  | (6.2380) | (3.0190) |
| *Size* | 0.1471^***^ | 0.1316^***^ |
|  | (17.1074) | (19.4592) |
| *Lev* | 0.1554^***^ | 0.1105^***^ |
|  | (4.1084) | (3.8429) |
| *ROA* | 0.2113^**^ | 0.4864^***^ |
|  | (2.1558) | (5.2977) |
| *Cashflow* | 0.1457 | 0.0511 |
|  | (1.3894) | (0.7594) |
| *Growth* | -0.0578^***^ | -0.0565^***^ |
|  | (-3.1368) | (-7.0944) |
| *Board* | 0.0502 | 0.0921^***^ |
|  | (1.1079) | (2.6060) |
| *Indep* | 0.0007 | 0.0010 |
|  | (0.4558) | (0.9090) |
| *Top1* | -0.0002 | -0.0006^*^ |
|  | (-0.3215) | (-1.7762) |
| *ListAge* | -0.0947^***^ | -0.0699^***^ |
|  | (-10.0420) | (-9.4920) |
| *Dual* | 0.0119 | 0.0211^*^ |
|  | (0.8427) | (1.8371) |
| *SOE* | 0.0858^***^ | 0.0719^***^ |
|  | (4.8841) | (5.8116) |
| _cons | -3.2101^***^ | -3.1034^***^ |
|  | (-12.5828) | (-19.4685) |
| Year fe | Yes | Yes |
| Ind fe | Yes | Yes |
| p-value | 0.000 | |
| *N* | 14539 | 20291 |
| r2 | 0.1820 | 0.1737 |

Note: ***p<0.01, **p<0.05, *p<0.1 are significant at the level of 1%, 5% and 10%, respectively.

*5.2.2 The influence of industry attributes*

Industry heterogeneity will lead to differences in corporate ethics and environmental awareness (Brammer et al., 2006), which may influence enterprises' choices of green innovation strategy. Therefore, industry heterogeneity may significantly affect the relationship between the positive tone of the annual report text and corporate green innovation. Compared with non-heavy polluting industries, heavy polluting industries will be subject to more stringent environmental supervision and pressure from environmental protection policies. On the other hand, green innovation will bring enterprises a better reputation and performance (Shi and Zhang, 2024), so the heavily polluting industries have a stronger incentive to implement green innovation. While non-heavy polluters often do not have a political advantage, when companies use a positive tone, they need to attract the attention of stakeholders and maintain a good corporate reputation for green innovation. Therefore, we predict that the positive relationship between positive text tone and green innovation is more obvious in non-heavy polluting enterprises.

According to the industry classification management directory of environmental protection verification of listed companies, we divided the sample enterprises into two sub-samples of heavy pollution enterprises and non-heavy pollution enterprises for heterogeneity test. The results in Table 11 show that the positive tone of the annual report text in non-heavy polluting industries is significantly and positively correlated with green innovation. In contrast, in heavy-polluting industries, *Tone*'s regression coefficient is positive but insignificant. When we further used the Bootstrap sampling method to test the significance of coefficient differences between groups again, the corresponding P value was 0.030, indicating that the positive impact of the positive tone of annual report text on enterprise green innovation was more significant in non-heavy pollution enterprises. This result further confirms the information increment effect of positive text intonation. (**Line 385-407 on page20)**

**Table 11	The influence of industry attributes**

| Variable | *Green* | |
| --- | --- | --- |
|  | (1)  Heavy-polluting industries | (2)  Non-heavy polluting industries |
| *Tone* | 0.9926^**^ | 1.8604^***^ |
|  | (2.2232) | (6.3929) |
| *Size* | 0.1251^***^ | 0.1388^***^ |
|  | (15.3121) | (21.4571) |
| *Lev* | -0.1300^***^ | 0.2124^***^ |
|  | (-2.8428) | (8.0826) |
| *ROA* | 0.0166 | 0.4077^***^ |
|  | (0.1184) | (5.6243) |
| *Cashflow* | 0.3876^***^ | 0.0133 |
|  | (3.4582) | (0.1992) |
| *Growth* | -0.0371^**^ | -0.0586^***^ |
|  | (-2.3156) | (-6.6983) |
| *Board* | 0.2627^***^ | 0.0264 |
|  | (4.9257) | (0.8153) |
| *Indep* | 0.0048^**^ | -0.0002 |
|  | (2.5740) | (-0.2030) |
| *Top1* | 0.0007 | -0.0007^**^ |
|  | (1.2346) | (-2.0890) |
| *ListAge* | -0.0736^***^ | -0.0818^***^ |
|  | (-6.3576) | (-12.1831) |
| *Dual* | -0.0440^***^ | 0.0320^***^ |
|  | (-2.7101) | (3.0687) |
| *SOE* | 0.1037^***^ | 0.0671^***^ |
|  | (5.4438) | (5.6458) |
| _cons | -3.0160^***^ | -3.0908^***^ |
|  | (-10.8181) | (-20.1513) |
| Year fe | Yes | Yes |
| Ind fe | Yes | Yes |
| p-value | 0.030 | |
| *N* | 7892 | 26938 |
| r2 | 0.1411 | 0.1861 |

Note: ***p<0.01, **p<0.05, *p<0.1 are significant at the level of 1%, 5% and 10%, respectively. ''

**13. Comment:** Results from the inter-group variance tests should be included in the heterogeneity analysis.

**13. Response:** Thank you for your suggestion, according to your suggestion, we have put the results of the inter-group difference test in Table 10 and Table 11. The revised Table 10 and Table 11 are as follows:

**Table 10	The influence of economic policy uncertainty**	(**Line 382on page19)**

| Variable | *Green* | |
| --- | --- | --- |
|  | (1)  *EPU_H* | (2)  *EPU_L* |
| *Tone* | 2.5714^***^ | 0.9124^***^ |
|  | (6.2380) | (3.0190) |
| *Size* | 0.1471^***^ | 0.1316^***^ |
|  | (17.1074) | (19.4592) |
| *Lev* | 0.1554^***^ | 0.1105^***^ |
|  | (4.1084) | (3.8429) |
| *ROA* | 0.2113^**^ | 0.4864^***^ |
|  | (2.1558) | (5.2977) |
| *Cashflow* | 0.1457 | 0.0511 |
|  | (1.3894) | (0.7594) |
| *Growth* | -0.0578^***^ | -0.0565^***^ |
|  | (-3.1368) | (-7.0944) |
| *Board* | 0.0502 | 0.0921^***^ |
|  | (1.1079) | (2.6060) |
| *Indep* | 0.0007 | 0.0010 |
|  | (0.4558) | (0.9090) |
| *Top1* | -0.0002 | -0.0006^*^ |
|  | (-0.3215) | (-1.7762) |
| *ListAge* | -0.0947^***^ | -0.0699^***^ |
|  | (-10.0420) | (-9.4920) |
| *Dual* | 0.0119 | 0.0211^*^ |
|  | (0.8427) | (1.8371) |
| *SOE* | 0.0858^***^ | 0.0719^***^ |
|  | (4.8841) | (5.8116) |
| _cons | -3.2101^***^ | -3.1034^***^ |
|  | (-12.5828) | (-19.4685) |
| Year fe | Yes | Yes |
| Ind fe | Yes | Yes |
| p-value | 0.000 | |
| *N* | 14539 | 20291 |
| r2 | 0.1820 | 0.1737 |

Note: ***p<0.01, **p<0.05, *p<0.1 are significant at the level of 1%, 5% and 10%, respectively.

**Table 11	The influence of industry attributes** (**Line 407on page20-21)**

| Variable | *Green* | |
| --- | --- | --- |
|  | (1)  Heavy-polluting industries | (2)  Non-heavy polluting industries |
| *Tone* | 0.9926^**^ | 1.8604^***^ |
|  | (2.2232) | (6.3929) |
| *Size* | 0.1251^***^ | 0.1388^***^ |
|  | (15.3121) | (21.4571) |
| *Lev* | -0.1300^***^ | 0.2124^***^ |
|  | (-2.8428) | (8.0826) |
| *ROA* | 0.0166 | 0.4077^***^ |
|  | (0.1184) | (5.6243) |
| *Cashflow* | 0.3876^***^ | 0.0133 |
|  | (3.4582) | (0.1992) |
| *Growth* | -0.0371^**^ | -0.0586^***^ |
|  | (-2.3156) | (-6.6983) |
| *Board* | 0.2627^***^ | 0.0264 |
|  | (4.9257) | (0.8153) |
| *Indep* | 0.0048^**^ | -0.0002 |
|  | (2.5740) | (-0.2030) |
| *Top1* | 0.0007 | -0.0007^**^ |
|  | (1.2346) | (-2.0890) |
| *ListAge* | -0.0736^***^ | -0.0818^***^ |
|  | (-6.3576) | (-12.1831) |
| *Dual* | -0.0440^***^ | 0.0320^***^ |
|  | (-2.7101) | (3.0687) |
| *SOE* | 0.1037^***^ | 0.0671^***^ |
|  | (5.4438) | (5.6458) |
| _cons | -3.0160^***^ | -3.0908^***^ |
|  | (-10.8181) | (-20.1513) |
| Year fe | Yes | Yes |
| Ind fe | Yes | Yes |
| p-value | 0.030 | |
| *N* | 7892 | 26938 |
| r2 | 0.1411 | 0.1861 |

Note: ***p<0.01, **p<0.05, *p<0.1 are significant at the level of 1%, 5% and 10%, respectively.

The above contents are our responses to your valuable questions or suggestions. Your high-quality suggestions are of great help in improving the quality of our paper. Special thanks to you for your good comments!

**Reviewer # 3:**

**1. Comment:** The number of green patent applications is a typical ordered count data, which does not obey the positive distribution. I do not recommend that authors use +1 and logarithmic data processing methods. I prefer to use Poisson regression or panel negative binomial regression.

**1. Response:** Thank you for pointing out this question. After descriptive statistics, we found that the green innovation data was obviously skewed to the right. Therefore, we followed the practice of previous studies and processed the green innovation data with logarithm after +1. Meanwhile, in the robustness test, according to your suggestion, we also used Poisson regression and panel negative binomial regression to conduct regression test again, and the research conclusion remained unchanged.

**2. Comment:** I strongly recommend that the sample data be updated to 2022. In the past two years, China's capital market has continued to improve, while the digital transformation of enterprises has become more mature. These reasons may have implications for green innovation.

**2. Response:** Thank you for your suggestions. According to your suggestion, we have updated the sample data to 2022.

**3. Comment:** The study did not consider causal inference. Note that PSM does not solve the endogeneity problem. Regression using a one-stage lag of the explained variable is also not a causal inference method. I recommend that authors adopt the tool variable and Heckman two-step method.

**3. Response:** Thank you for your suggestions. According to your suggestion, we have adopted the instrumental variable method to solve the endogeneity problem. The newly added instrumental variable method section is as follows:

''*4.5.6 Instrumental variable method*

Considering that there may be a reverse causal relationship between the positive tone of annual report texts and green innovation, following Li and Jiang (2020), we select the mean value of the positive tone of texts in the same year and industry as the instrumental variable (*Tone_m*) and adopt the two-stage least squares method (IV-2SLS).

The regression results are shown in Table 8. The test results of weak instrumental variables show that the F value is 3054.94, which is significantly greater than the empirical value 10. The instrumental variables satisfy the correlation and are strong instrumental variables. The regression coefficient of *Tone* is still significantly positive, which once again verifies the positive tone of the annual report text to promote enterprises' green innovation.'' (**Line 309-319 on page15**)

**Table 8	Instrumental variable method**

| Variable | *Green* | |
| --- | --- | --- |
|  | (1) | (2) |
| *Tone_m* | 1.3852^**^ |  |
|  | (2.0597) |  |
| *Tone* |  | 1.7996^**^ |
|  |  | (2.0609) |
| *Size* | 0.1372^***^ | 0.1364^***^ |
|  | (33.8924) | (33.2899) |
| *Lev* | 0.1407^***^ | 0.1352^***^ |
|  | (5.6741) | (5.4336) |
| *ROA* | 0.4021^***^ | 0.3346^***^ |
|  | (5.5959) | (4.2046) |
| *Cashflow* | 0.0840 | 0.0920 |
|  | (1.3763) | (1.5049) |
| *Growth* | -0.0509^***^ | -0.0554^***^ |
|  | (-5.3560) | (-5.6942) |
| *Board* | 0.0765^***^ | 0.0742^***^ |
|  | (3.1126) | (3.0151) |
| *Indep* | 0.0009 | 0.0009 |
|  | (1.1232) | (1.1035) |
| *Top1* | -0.0005^*^ | -0.0005^*^ |
|  | (-1.7100) | (-1.6592) |
| *ListAge* | -0.0812^***^ | -0.0792^***^ |
|  | (-13.9104) | (-13.2109) |
| *Dual* | 0.0177^**^ | 0.0171^*^ |
|  | (2.0134) | (1.9424) |
| *SOE* | 0.0814^***^ | 0.0763^***^ |
|  | (8.1450) | (7.2863) |
| _cons | -2.9496^***^ | -2.9495^***^ |
|  | (-9.2057) | (-9.2108) |
| Year fe | Yes | Yes |
| Ind fe | Yes | Yes |
| F-value | 3054.94 | |
| *N* | 34799 | 34799 |
| r2 | 0.1754 | 0.1764 |

Note: ***p<0.01, **p<0.05, *p<0.1 are significant at the level of 1%, 5% and 10%, respectively.''

**4. Comment:** Baseline regression. I strongly recommend that the authors adequately compare the results of this study with existing literature. For the researchers, we are eager to know whether this manuscript is the first to discover the positive effect of positive tone in annual reports on green innovation, or whether it is a re-examination of the existing literature.

**4. Response:** According to your suggestion, we have adopted the results of this study with existing literature. The revised part is as follows:

'' The empirical results show that the annual report text's positive tone plays an "information incremental effect"(Xie and Lin,2015; Jiang et al, 2019; Wang et al.2024), and positively promotes the green innovation behavior of enterprises, that is, H1a was verified. This conclusion is consistent with the relevant research conclusion of Yu (2022).'' (**Line 245-247 on page10**)

**5. Comment:** Study limitations and future prospects are suggested to be supplemented.

**5. Response:** Thank you for pointing out this issue, and according to your suggestion, we added a section on limitations and perspectives as follows:

'' This study has several limitations, including (i)the positive tone of the text is limited to management discussion and analysis in the annual report, (ii) other measures of green innovation are not used, (iii) a simple regression model is used, and (iv) the influence of external governance mechanisms on the study is not considered. Therefore, future research may use more channels of annual report text tone, use other green innovation measures, apply other suitable models, and consider the impact of multiple background factors on the research (e.g., media coverage, institutional investors, and independent directors).'' (**Line 461-467 on page24**)

**6. Comment:** I suggest that the author add a research flow chart to the introduction.

**6. Response:** Thank you for pointing out this issue, and according to your suggestion, we have added a research flow chart to the introduction. The research flow chart is as follows:

'' The research flow chart of this paper is shown in Figure 1. '' (**Line 83on page3**)

**Figure 1. the research flow chart**

We appreciate for Editors and Reviewers’ warm work earnestly, and we tried our best to improve the manuscript and made some changes in the manuscript. We hope that the correction will meet with approval.

Thanks again for your helpful comments and suggestions! Best wishes to you!
