# Supplementary material for: Annual report text’s positive tone and corporate green innovation: Evidence from China
Source: PLoS One. 2024 Jul 29;19(7):e0306184. doi: 10.1371/journal.pone.0306184 (PMC11285926; doi:10.1371/journal.pone.0306184)
Supplement: Attachment — Submitted filename: Response to Reviewers.docx [file pone.0306184.s003.docx]

Dear Editors and Reviewers:

Thank you very much for your comments and professional advice concerning our manuscript entitled ''**Annual report text's positive tone and corporate green innovation: Evidence from China**'' (**ID: PONE-D-24-12298**). Those comments are all valuable and helpful for revising and improving our paper. We have studied the comments carefully and have revised our manuscript. Words in **blue color** are the changes we have made in the revised manuscript. Now we respond to your comments **point by point** and highlight the changes in the revised manuscript. We sincerely hope that you find our responses and modifications satisfactory. The responses to your comments are listed as follows:

**Reviewer # 1:**

**1.Comment:** Please pay attention to the formatting of the formula. For instance, in formula (2) should not be on a separate line, and formula (3) should be centered.

**1. Response:** Thank you for your comments and suggestions, we have modified formula (2) to ensure that the format is standardized. the revised Equation 2 as follows:

${Green}_{i,t}=\alpha_{0}+\alpha_{1}{Tone}_{i,t}+\alpha_{j}\sum{Controls}_{i,t}+\sum{Year}_{i,t}+\sum{Ind}_{i,t}+\varepsilon_{i,t}$(2)

(**Line 209 on page8)**

The above contents are our responses to your valuable questions or suggestions. Thank you very much for your patience! Your patience and high-quality advice are helpful to improve our paper.

**Reviewer # 2:**

**1. Comment:**  The inter-group variance tests for heterogeneity analysis are missing. Where have they been conducted?

**1. Response:** Thank you very much for asking this question, but we have to explain this, for the coefficient difference between groups, we've done this in the original manuscript using the Bootstrap sampling method to test the significance of inter-group coefficient differences. Moreover, it is also explained in the paper, including the method and p-value, and the P-value is also shown in the table. The specific contents of the inter-group coefficient difference test are as follows:

'' However, when we further used the Bootstrap sampling method to test the significance of inter-group coefficient differences, the corresponding P-value was 0.000, indicating that when the economic policy uncertainty was higher, the positive tone of the annual report had a more obvious role in promoting corporate green innovation. This test result further confirmed the information incremental effect of positive tone of the text. '' (**Line 384-486 on page19)**

**Table 11	The influence of economic policy uncertainty**

| Variable | *Green* | |
| --- | --- | --- |
|  | (1)  *EPU_H* | (2)  *EPU_L* |
| *Tone* | 2.5714^***^ | 0.9124^***^ |
|  | (6.2380) | (3.0190) |
| *Size* | 0.1471^***^ | 0.1316^***^ |
|  | (17.1074) | (19.4592) |
| *Lev* | 0.1554^***^ | 0.1105^***^ |
|  | (4.1084) | (3.8429) |
| *ROA* | 0.2113^**^ | 0.4864^***^ |
|  | (2.1558) | (5.2977) |
| *Cashflow* | 0.1457 | 0.0511 |
|  | (1.3894) | (0.7594) |
| *Growth* | -0.0578^***^ | -0.0565^***^ |
|  | (-3.1368) | (-7.0944) |
| *Board* | 0.0502 | 0.0921^***^ |
|  | (1.1079) | (2.6060) |
| *Indep* | 0.0007 | 0.0010 |
|  | (0.4558) | (0.9090) |
| *Top1* | -0.0002 | -0.0006^*^ |
|  | (-0.3215) | (-1.7762) |
| *ListAge* | -0.0947^***^ | -0.0699^***^ |
|  | (-10.0420) | (-9.4920) |
| *Dual* | 0.0119 | 0.0211^*^ |
|  | (0.8427) | (1.8371) |
| *SOE* | 0.0858^***^ | 0.0719^***^ |
|  | (4.8841) | (5.8116) |
| _cons | -3.2101^***^ | -3.1034^***^ |
|  | (-12.5828) | (-19.4685) |
| Year fe | Yes | Yes |
| Ind fe | Yes | Yes |
| p-value | 0.000 | |
| *N* | 14539 | 20291 |
| r2 | 0.1820 | 0.1737 |

'' When we further used the Bootstrap sampling method to test the significance of coefficient differences between groups again, the corresponding P value was 0.030, indicating that the positive impact of the positive tone of annual report text on enterprise green innovation was more significant in non-heavy pollution enterprises. ''(**Line 409-410 on page21)**

**Table 12	The influence of industry attributes**

| Variable | *Green* | |
| --- | --- | --- |
|  | (1)  Heavy-polluting industries | (2)  Non-heavy polluting industries |
| *Tone* | 0.9926^**^ | 1.8604^***^ |
|  | (2.2232) | (6.3929) |
| *Size* | 0.1251^***^ | 0.1388^***^ |
|  | (15.3121) | (21.4571) |
| *Lev* | -0.1300^***^ | 0.2124^***^ |
|  | (-2.8428) | (8.0826) |
| *ROA* | 0.0166 | 0.4077^***^ |
|  | (0.1184) | (5.6243) |
| *Cashflow* | 0.3876^***^ | 0.0133 |
|  | (3.4582) | (0.1992) |
| *Growth* | -0.0371^**^ | -0.0586^***^ |
|  | (-2.3156) | (-6.6983) |
| *Board* | 0.2627^***^ | 0.0264 |
|  | (4.9257) | (0.8153) |
| *Indep* | 0.0048^**^ | -0.0002 |
|  | (2.5740) | (-0.2030) |
| *Top1* | 0.0007 | -0.0007^**^ |
|  | (1.2346) | (-2.0890) |
| *ListAge* | -0.0736^***^ | -0.0818^***^ |
|  | (-6.3576) | (-12.1831) |
| *Dual* | -0.0440^***^ | 0.0320^***^ |
|  | (-2.7101) | (3.0687) |
| *SOE* | 0.1037^***^ | 0.0671^***^ |
|  | (5.4438) | (5.6458) |
| _cons | -3.0160^***^ | -3.0908^***^ |
|  | (-10.8181) | (-20.1513) |
| Year fe | Yes | Yes |
| Ind fe | Yes | Yes |
| p-value | 0.030 | |
| *N* | 7892 | 26938 |
| r2 | 0.1411 | 0.1861 |

**2. Comment:** The presence of micro-level data does not justify overlooking macro-level influences. Many micro-studies control for macroeconomic variables.

**2. Response:** Thank you very much for your questions and suggestions. According to your suggestions, after adding macroeconomic variables, we re-regression model (2), the research results remain unchanged, and the new contents are as follows:

'' *4.5.7 Adding macro variables*

To make the results more robust, we added a macroeconomic variable, the value-added rate of gross domestic product (*GDP*), as a control variable. The regression results after adding macroeconomic variables are shown in Table9. The regression coefficient of *Tone* is 1.6409, which is significant at the 1% level, indicating that our main regression results are still robust. '''(**Line 320-325 on page16)**

**Table 9	Robust test after adding macro variables**

| Variable | *Green* |
| --- | --- |
|  | (1) |
| *Tone* | 1.6409^***^ |
|  | (6.6681) |
| *Size* | 0.1360^***^ |
|  | (25.5848) |
| *Lev* | 0.1385^***^ |
|  | (6.0557) |
| *ROA* | 0.3509^***^ |
|  | (5.4223) |
| *Cashflow* | 0.0925 |
|  | (1.5993) |
| *Growth* | -0.0562^***^ |
|  | (-7.3296) |
| *Board* | 0.0703^**^ |
|  | (2.5238) |
| *Indep* | 0.0008 |
|  | (0.9061) |
| *Top1* | -0.0005^*^ |
|  | (-1.7102) |
| *ListAge* | -0.0813^***^ |
|  | (-13.7965) |
| *Dual* | 0.0166^*^ |
|  | (1.8508) |
| *SOE* | 0.0789^***^ |
|  | (7.7456) |
| *GDP* | -0.0160^***^ |
|  | (-6.4435) |
| _cons | -2.7560^***^ |
|  | (-21.4502) |
| Year fe | Yes |
| Ind fe | Yes |
| *N* | 34664 |
| r2 | 0.1769 |

**3. Comment:** Equations (1), (2), and (3) are not displayed on the same line. The author needs to approach the manuscript revision with greater seriousness. **✅**

**3. Response:** Thank you for your comments and suggestions, to ensure that equations (1), (2) and (3) have a consistent format, we have modified Equation 2. the revised Equation 2 as follows:

${Green}_{i,t}=\alpha_{0}+\alpha_{1}{Tone}_{i,t}+\alpha_{j}\sum{Controls}_{i,t}+\sum{Year}_{i,t}+\sum{Ind}_{i,t}+\varepsilon_{i,t}$(2)

(**Line 209 on page8)**

**4. Comment:** The author has not amended the following incomplete sentence which lacks a predicate: "Section 2 literature review, theoretical analysis, and hypothesis. Section 5 mechanism test and heterogeneity analysis." Is the author taking the review process lightly?

**4. Response:** We feel so sorry for the mistakes in the manuscript and inconvenience they caused in your reading. The language in our revised manuscript has been professionally edited and verified by a native English speaker. Thank you very much for your precious comments. We take every question you raise very seriously and try our best to revise the manuscript. Thank you very much for the time and effort you have put into the manuscript. We really hope that the flow and language level have been substantially improved.

**5. Comment:** I suggest the author thoroughly proofread the manuscript before resubmitting！

**5. Response:** Thank you very much for your suggestion. We have carried out a comprehensive examination of the manuscript and hope to get your approval.

The above contents are our responses to your valuable questions or suggestions. Your high-quality suggestions are of great help in improving the quality of our paper. Special thanks to you for your good comments!

**Reviewer # 3:**

**1. Comment:** The author did a good job of solving my earlier doubts. On the whole, I am satisfied with the revised manuscript. Before accepting this manuscript, however, I recommend that the authors intensify the literature review. I highly recommend adding references from the last three years to highlight the timeliness of the manuscript. I have listed some of the literature, and authors can decide whether they need to cite them based on the relevance of the research.
Hu, J., Hu, M., & Zhang, H. (2023). Has the construction of ecological civilization promoted green technology innovation?. Environmental Technology & Innovation, 29, 102960.
Bai, D.B., Hu, J., Irfan, M., & Hu, M.J. (2023). Unleashing the impact of ecological civilization pilot policies on green technology innovation: Evidence from a novel SC-DID model. Energy Economics, 106813.
Han, Y., Li, Z., Feng, T., Qiu, S., Hu, J., Yadav, K. K., & Obaidullah, A. J. (2024). Unraveling the impact of digital transformation on green innovation through microdata and machine learning. Journal of Environmental Management, 354, 120271.
Shen, Y., Zhang, X. (2023). Intelligent manufacturing, green technological innovation and environmental pollution. Journal of Innovation & Knowledge 8(3), 100384.
**1. Response:** Thank you very much for your suggestion, we have added to the literature according to your suggestion. The changes are as follows:

'' Green innovation, as a combination of the two new development concepts of "green" and "innovation," has been widely recognized and supported by the public (Hu et al., 2023; Bai et al., 2023). However, compared with the general technological innovation, the natural high risk, uncertainty, and long-term cyclical characteristics of green innovation will have a superimposed impact on the business risk of enterprises, which may lead to the lack of internal drive for green innovation (Han et al., 2024; Shen and Zhang, 2023). '' (**Line 108, 111-112 on page 4**)

**2. Comment:** Finally, I suggest replacing the table in Table 3 with a picture. Thermodynamic coefficient is recommended.

**2. Response:** Thank you for your suggestions. According to your suggestion, we have converted Table 3 into a picture, as follows: (**Line232 on page9**)

We appreciate for Editors and Reviewers’ warm work earnestly, and we tried our best to improve the manuscript and made some changes in the manuscript. We hope that the correction will meet with approval.

Thanks again for your helpful comments and suggestions! Best wishes to you!
