# Supplementary material for: Annual report text’s positive tone and corporate green innovation: Evidence from China
Source: PLoS One. 2024 Jul 29;19(7):e0306184. doi: 10.1371/journal.pone.0306184 (PMC11285926; doi:10.1371/journal.pone.0306184)
Supplement: Attachment — Submitted filename: Response to Reviewers.docx [file pone.0306184.s004.docx]

Dear Editors and Reviewers:

Thank you very much for your comments and professional advice concerning our manuscript entitled ''**Annual report text's positive tone and corporate green innovation: Evidence from China**'' (**ID: PONE-D-24-12298**). Those comments are all valuable and helpful for revising and improving our paper. We have studied the comments carefully and have revised our manuscript. Words in **blue color** are the changes we have made in the revised manuscript. Now we respond to your comments **point by point** and highlight the changes in the revised manuscript. We sincerely hope that you find our responses and modifications satisfactory. The responses to your comments are listed as follows:

**Reviewer # 2:**

**1. Comment:**  The author still hasn't solved my problem. The sentence in lines 78-83 in the manuscript lacks the necessary verb, and I've already mentioned the same problem twice！

**1. Response:** Thank you very much for your careful guidance in pointing out the problem of grammatical errors in the article. I am very sorry for the trouble caused to your reading. We have made careful revisions to address the language issues raised and hope to have your approval. The revisions are as follows:

'' The remainder of this study is organized as follows: Section 2 reviews the extant literature, discusses the theoretical framework of hypotheses and the potential channel in detail. Section 3 outlines the definitions of data and variables, as well as the construction of models. Section 4 presents the results and discussion, addresses the issue of endogeneity and robustness tests, and investigates the potential channels. Section 5 conducts mechanism tests, heterogeneity analysis and economic effects tests. Section 6 summarizes the conclusions and discusses the insights, limitations and future perspectives. This paper's research flow chart is shown in Figure 1.''(**Line 78-84 on page3)**

The above contents are our responses to your valuable questions or suggestions. Your high-quality suggestions are of great help in improving the quality of our paper. Special thanks to you for your good comments!

**Reviewer # 3:**

**1. Comment:**  Correlation coefficient matrix can be used in heat maps. Display the picture in one visual and color, rather than simply replacing it with an image format.

**1. Response:** Thank you very much for your careful guidance. We have done the heat map as you suggested. We need to explain to you that we have not done a heat map before because the area of study has not seen such a map, which is usually presented in a table. So, we hope that the revised diagram meets your requirements. The revisions are as follows: (**Line 232 on page 9**)

**Figure 2 Correlation analysis heat map**

**2. Comment:**  Cite Chinese references as little as possible

**2. Response:** Thank you for your suggestions. According to your suggestion, we have deleted some Chinese literature.

**3. Comment:** I suggest that the authors supplement the statement that instrumental variables satisfy the principles of relevance and exclusivity. Why does it have such a strong exogeneity?

**3. Response:** Thank you for your suggestions. According to your suggestion, we provide a detailed representation of the correlation and exogeneity of the instrumental variables. The revisions are as follows:

''We adopt this idea of instrumental variables mainly for the following two reasons: Firstly, enterprises within the same industry have similar operation and business scope, and will be affected by the same market environment and regulatory rules. Therefore, the narrative logic and expression of the annual report texts of enterprises in the same industry tend to be more similar, presenting certain industry characteristics, which meets the relevance requirements of the instrumental variables. Secondly, the language characteristics of other enterprises' annual report texts do not have an impact on this enterprise's green innovation, which meets the requirement of exogeneity of instrumental variables. '' (**Line315-322 on page15**)

**4. Comment:** Whether the result reported in parentheses is a t statistic or a standard error needs to be explained in a footnote.

**4. Response:** Thank you for your suggestions. According to your suggestion, we have detailed notes underneath all the tables.

We appreciate for Editors and Reviewers’ warm work earnestly, and we tried our best to improve the manuscript and made some changes in the manuscript. We hope that the correction will meet with approval.

Thanks again for your helpful comments and suggestions! Best wishes to you!
